# The hidden burden of sarcopenia in inflammatory bowel disease: a strong predictor of complications

**DOI:** 10.3389/fgstr.2026.1960689

**Published:** 2026-10-28

**Authors:** Alessia Dalila Guarino, Giulio Calabrese, Maurizio Marra, Ersilia Nigro, Aurora Daniele, Antonio Rispo, Anna Testa, Olga Maria Nardone, Ludovica De Vito, Camilla Leo, Fabrizio Pasanisi, Fabiana Castiglione

**Affiliations:** 1 Gastroenterology, Department of Clinical Medicine and Surgery, University of Naples Federico II, Naples, Italy; 2 Department of Clinical Medicine and Surgery, University of Naples “Federico II”, Naples, Italy; 3 CEINGE-Biotechnologies Advances, Naples, Italy; 4 Department of Molecular and Biotechnological Medicine, University of Naples “Federico II”, Naples, Italy; 5 Department of Public Health, University of Naples Federico II, Naples, Italy

**Keywords:** Crohn ‘ s disease, IBD, predictor, sarcopenia, ulcerative colitis

## Abstract

**Background:**

Sarcopenia is an emerging predictor of adverse outcomes in inflammatory bowel disease (IBD). We assessed its prevalence and prognostic impact in a prospective cohort.

**Methods:**

Consecutive adults with IBD were prospectively enrolled (December 2023–August 2024). Sarcopenia was assessed using handgrip strength and bioelectrical impedance analysis (BIA) and patients were classified as non sarcopenic, with probable sarcopenia, or confirmed sarcopenia. Clinical worsening, IBD-related hospitalization, surgery, and infectious complications were prospectively assessed over 12 months.

**Results:**

We enrolled 113 IBD patients: 32 (28.3%) were non-sarcopenic, 73 (64.6%) had probable sarcopenia, and 8 (7.1%) had confirmed sarcopenia. Confirmed sarcopenia was associated with lower BMI, higher CRP levels, and a higher prevalence of high nutritional risk. During follow-up, clinical worsening occurred in 46.9%, 45.2%, and 87.5% of patients, respectively (p=0.074), while IBD-related hospitalization occurred in 6.2%, 11.0%, and 62.5% (p<0.001). After adjustment for baseline clinical activity, confirmed sarcopenia remained independently associated with IBD-related hospitalization (adjusted OR [aOR] 18.30, 95% CI 3.21–141.14; p<0.001). Confirmed sarcopenia was also independently associated with infectious complications after adjustment for advanced-therapy exposure (aOR 7.40, 95% CI 1.17–54.63; p=0.034). No independent association was observed between sarcopenia and clinical worsening after adjustment for age, baseline clinical activity, BMI, and CRP.

**Conclusions:**

In this prospective cohort of patients with IBD, confirmed sarcopenia was independently associated with an increased risk of IBD-related hospitalization and infectious complications over 12 months. These findings suggest that assessment of muscle strength and mass may provide clinically relevant prognostic information beyond conventional measures of disease activity and nutritional status.

## Introduction

Inflammatory bowel diseases (IBD), including Crohn’s disease (CD), ulcerative colitis (UC), and inflammatory bowel disease-unclassified (IBD-U), are chronic, immune-mediated disorders characterized by relapsing intestinal inflammation and a substantial systemic eliminate inflammatory burden (1). Beyond gastrointestinal involvement, IBD is increasingly recognized as a systemic disease affecting nutritional status, body composition, and overall metabolic health.

Malnutrition represents one of the most frequent extra-intestinal complications of IBD and is particularly prevalent in patients with CD, affecting up to 65–75% of cases (2–5). Its pathogenesis is multifactorial and involves reduced dietary intake, malabsorption, increased metabolic demands, and persistent inflammation. Consequently, nutritional impairment extends beyond body weight reduction and is characterized by profound alterations in body composition, including depletion of fat-free mass and skeletal muscle, together with micronutrient deficiencies (5–8).

Within this context, sarcopenia has emerged as a clinically relevant condition in patients with IBD. According to the revised European Working Group on Sarcopenia in Older People (EWGSOP2), sarcopenia is defined as a progressive loss of skeletal muscle strength and muscle mass, with muscle strength representing the primary parameter for case identification (4). Although traditionally considered an age-related disorder, sarcopenia is increasingly recognized as secondary to chronic inflammatory diseases and may affect young adults with IBD (4, 9).

The mechanisms underlying sarcopenia in IBD are complex and multifactorial. Chronic inflammation promotes muscle protein catabolism through the activation of pro-inflammatory cytokines, including tumor necrosis factor-α (TNF-α), interferon-γ (IFN-γ), interleukin (IL)-1, IL-6, and IL-12, resulting in impaired muscle protein synthesis and accelerated proteolysis. In parallel, inadequate nutrient intake, intestinal malabsorption, increased energy expenditure, and reduced physical activity further contribute to progressive muscle wasting. In recent years, growing evidence has also supported the existence of a gut–muscle axis, whereby intestinal inflammation, gut microbiota alterations, and nutritional impairment synergistically contribute to skeletal muscle dysfunction (3, 10–12).

The reported prevalence of sarcopenia in IBD ranges from approximately 15% to 40%, largely reflecting differences in study populations, diagnostic criteria, and methods used to assess muscle mass and function (12–14). Nevertheless, accumulating evidence indicates that sarcopenia is associated with poorer clinical outcomes, including an increased risk of hospitalization, postoperative complications, reduced response to biological therapies, impaired quality of life, and greater healthcare utilization (12–15).

Beyond skeletal muscle abnormalities, alterations in adipose tissue biology may also contribute to disease progression. Adipose tissue is increasingly recognized as an active endocrine organ capable of modulating inflammatory and metabolic pathways through the secretion of adipokines (16). Among these, adiponectin has attracted considerable interest because of its anti-inflammatory and metabolic properties. Altered circulating adiponectin concentrations have been associated with abnormalities in body composition, muscle dysfunction, and chronic inflammatory disorders (16). Furthermore, recent evidence has demonstrated a significant association between adiponectin, phase angle, and sarcopenia in patients with CD, suggesting that adiponectin may represent a potential biomarker linking chronic inflammation and muscle impairment in IBD (17).

Collectively, these observations highlight the close interplay between chronic inflammation, malnutrition, adipose tissue dysfunction, and skeletal muscle loss in patients with IBD. Early nutritional screening, comprehensive body composition assessment, and timely identification of muscle impairment may therefore play a pivotal role in improving risk stratification and optimizing patient management.

Accordingly, the present prospective study aimed to determine the prevalence of sarcopenia in a well-characterized cohort of patients with IBD and to evaluate its association with subsequent adverse clinical outcomes during longitudinal follow-up.

## Methods

### Study design and population

This prospective observational study was conducted at the Inflammatory Bowel Disease Unit of the University Hospital Federico II, Naples, Italy, and is reported in accordance with the Strengthening the Reporting of Observational Studies in Epidemiology (STROBE) guidelines (18). The study protocol was approved by the local Ethics Committee (Protocol No. 19/23; September 26, 2023), and all participants provided written informed consent before enrolment.

Consecutive adult patients with a confirmed diagnosis of Crohn’s disease (CD) or ulcerative colitis (UC) attending scheduled outpatient visits between December 2023 and August 2024 were prospectively enrolled, irrespective of disease activity or ongoing medical treatment.

Eligibility criteria included age ≥18 years, a confirmed diagnosis of CD or UC based on established clinical, endoscopic, radiological, and histological criteria, regular follow-up at the study center, and complete clinical and laboratory data.

Patients were excluded if they had active malignancy, advanced chronic liver disease, end-stage renal disease, neuromuscular disorders potentially affecting muscle mass or function, pregnancy, or severe acute infection at the time of enrolment.

### Clinical and biochemical assessment

Demographic, clinical, laboratory, and treatment-related data were prospectively collected at enrolment using standardized case report forms.

Baseline variables included age, sex, body mass index (BMI), disease duration, previous IBD-related surgery, disease phenotype according to the Montreal classification, ongoing medical therapy, and previous biologic treatment failure.

For patients with CD, disease location (L1–L3) and behavior (B1–B3) were recorded, whereas disease extent in UC was classified as E1–E3 according to the Montreal classification.

Clinical disease activity was assessed using the Harvey–Bradshaw Index (HBI) for CD and the partial Mayo (pMayo) score for UC. Active disease was defined as HBI ≥5 for CD and pMayo ≥2 for UC.

When available, endoscopic activity was assessed using the Simple Endoscopic Score for Crohn’s Disease (SES-CD) and the Mayo Endoscopic Subscore (MES). Active endoscopic disease was defined as SES-CD ≥3 or MES ≥2.

Bowel ultrasound examinations were performed according to standardized criteria (1). Ultrasonographic activity was defined by bowel wall thickness >3 mm and increased vascularity on color Doppler imaging.

Extraintestinal manifestations were recorded and categorized as articular, cutaneous, or ocular.

Health-related quality of life and disability were evaluated using validated questionnaires, including the Short Inflammatory Bowel Disease Questionnaire (SIBDQ), the Inflammatory Bowel Disease Disability Index (IBD-DI), and the IBD Disk. Moderate-to-severe disability was defined as an IBD-DI score ≥40 (19).

Clinical outcomes were prospectively assessed by the treating IBD physician during scheduled follow-up visits and complemented by review of electronic medical records, according to predefined study criteria.

Nutritional status was assessed using BMI, while the risk of malnutrition was evaluated with the Malnutrition Universal Screening Tool (MUST). A MUST score ≥2 indicated a high risk of malnutrition (20, 21). Adherence to the Mediterranean diet was assessed using the MEDI-Lite questionnaire, with a score ≥9 indicating high adherence (22).

Serum C-reactive protein (CRP), albumin, and adiponectin concentrations were measured from fasting blood samples collected at enrolment. Adiponectin was quantified using a commercially available enzyme-linked immunosorbent assay (ELISA), according to the manufacturer’s instructions, as previously described (17).

### Definition of sarcopenia

Sarcopenia was diagnosed according to the revised European Working Group on Sarcopenia in Older People (EWGSOP2) criteria (4).

Muscle strength was evaluated by handgrip dynamometry using a calibrated handheld dynamometer. Measurements were performed according to standardized procedures, and the highest value obtained from repeated attempts was recorded. Low muscle strength was defined as handgrip strength <27 kg in men and <16 kg in women. Patients with reduced muscle strength were classified as having probable sarcopenia.

Muscle mass was assessed by bioelectrical impedance analysis (BIA). Appendicular skeletal mass (ASM) was calculated using the Sergi equation (23) and adjusted by height squared (kg/m²) to derive the appendicular skeletal muscle mass index (ASMI). Low muscle mass was defined as an ASMI <7.0 kg/m² in men and <5.5 kg/m² in women.

Confirmed sarcopenia was defined by the coexistence of low muscle strength and low muscle mass, in accordance with EWGSOP2 recommendations (4). Because physical performance was not systematically evaluated, severe sarcopenia could not be classified.

### Handgrip strength assessment

Handgrip strength (HGS) was assessed using a JAMAR dynamometer (Roylan, UK). Bedridden patients were instructed to position their arms parallel to the trunk, grasp the dynamometer, and exert the maximum possible force with each hand. Measurements were performed three times for each hand, with a 1-min interval between consecutive measurements to minimize fatigue. In patients receiving intravenous therapy or presenting other limitations that precluded bilateral assessment, HGS was measured on the unaffected or available side only. Both the mean and the maximum values of the three measurements were recorded in kilograms (kg). Low muscle strength was defined as HGS <27 kg in men and <16 kg in women, according to the EWGSOP2 cut-off values.

### Bioelectrical impedance analysis

Bioelectrical impedance analysis (BIA) was performed using a Human Im Touch device (DS Medica S.r.l., Milan, Italy) at a frequency of 50 kHz under standardized conditions. Participants were instructed to abstain from alcohol consumption, smoking, and strenuous physical activity for 24 h before the assessment. Measurements were performed after an overnight fast of >8 h and with an empty bladder; in women of reproductive age, assessments were performed during the follicular phase whenever applicable. Room temperature was maintained at 23–25 °C.

Before measurement, participants remained in the supine position for at least 10 min. After cleaning the skin surface, they were positioned supine with the upper and lower limbs slightly abducted to avoid contact between the extremities and the trunk. A standard tetrapolar technique was used with disposable adhesive electrodes (FIAB S.r.l., Florence, Italy). Measuring electrodes were positioned on the anterior surface of the wrist and ankle, whereas current-injecting electrodes were placed on the dorsal surface of the hand and foot.

### Outcomes and follow-up

The primary objective was to evaluate the occurrence of adverse clinical outcomes during the 12-month follow-up period.

Prospectively recorded outcomes included clinical worsening, IBD-related hospitalization, IBD-related surgery, infectious complications, and treatment modification.

Clinical worsening was defined as new-onset or worsening IBD-related symptoms requiring treatment escalation or initiation of systemic corticosteroids, and/or objective evidence of increased disease activity based on validated clinical or instrumental assessment.

IBD-related hospitalization was defined as any hospital admission attributable to disease activity or disease-related complications. IBD-related surgery included any surgical intervention performed for IBD-related indications.

Infectious complications were defined as infections requiring antimicrobial therapy and/or hospitalization.

Treatment modification was defined as treatment escalation, optimization, or switching because of inadequate response, secondary loss of response, or drug intolerance.

Clinical outcomes were prospectively collected during scheduled outpatient visits and through review of electronic medical records.

### Statistical analysis

Continuous variables are reported as mean ± standard deviation (SD) or median with interquartile range (IQR), as appropriate. Categorical variables are presented as absolute numbers and percentages.

Continuous variables were compared across the three sarcopenia groups using one-way analysis of variance (ANOVA) or the Kruskal–Wallis test, according to data distribution. Categorical variables were analyzed using Pearson’s χ² test or Fisher’s exact test, as appropriate. When an overall comparison was statistically significant, pairwise *post-hoc* comparisons were performed where appropriate, with p-values adjusted for multiple comparisons using the Holm method.

Potential multicollinearity among covariates included in the main multivariable model was assessed using the generalized variance inflation factor (GVIF).

Given the limited number of events for some outcomes and the potential for sparse-data bias and separation, univariable and multivariable associations were assessed using Firth penalized logistic regression. Covariates were prespecified on the basis of clinical relevance rather than selected through data-driven stepwise procedures. For clinical worsening, the multivariable model included sarcopenia status, age, baseline clinical activity, BMI, and CRP. Given the smaller number of events, more parsimonious models were used for hospitalization and infectious complications: hospitalization was adjusted for baseline clinical activity, whereas infectious complications were adjusted for baseline advanced-therapy exposure.

Results are reported as odds ratios (ORs) with 95% confidence intervals (95% CIs). All statistical tests were two-sided, and a p value <0.05 was considered statistically significant.

Model discrimination was assessed using the area under the receiver operating characteristic curve (AUC), with 95% confidence intervals calculated using the DeLong method.

No *a priori* sample-size calculation was performed; the study included all consecutive eligible patients enrolled during the predefined recruitment period.

Statistical analyses were performed using RStudio (version 2025.09.1 + 401; R Foundation for Statistical Computing, Vienna, Austria).

## Results

### Study population and baseline characteristics

During the study period, 142 patients were assessed for eligibility. Twenty-nine patients (20.4%) were excluded because of incomplete sarcopenia assessment (missing handgrip strength and/or bioelectrical impedance analysis data, *n* = 16), incomplete clinical or laboratory data (*n* = 8), failure to meet the inclusion criteria (*n* = 3), or withdrawal of consent (*n* = 2). The remaining 113 patients were included in the final analysis (**Figure 1**).

**Figure 1:**
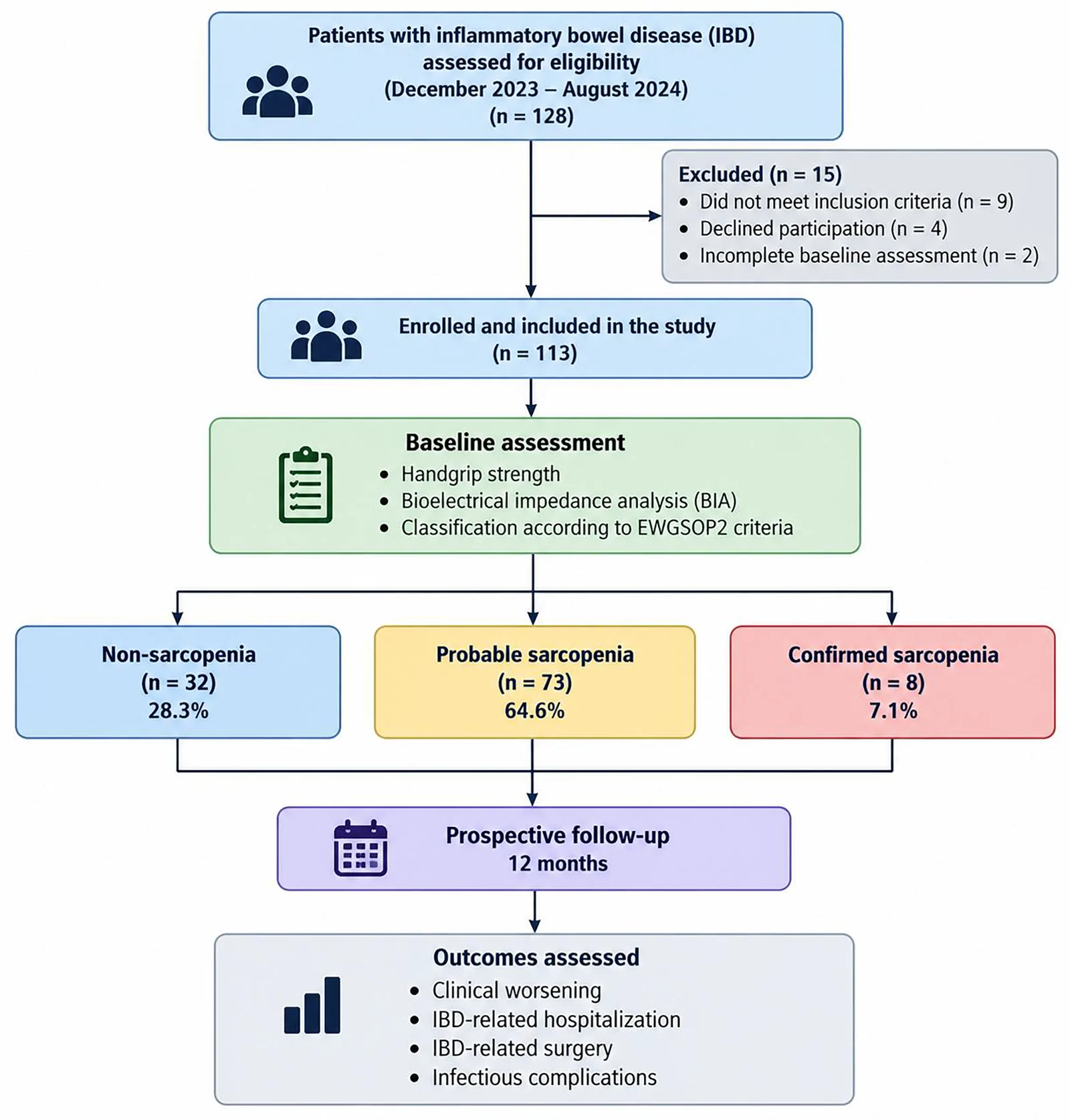
Study flow.

According to the EWGSOP2 criteria, 32 patients (28.3%) were classified as non-sarcopenic, 73 (64.6%) as having probable sarcopenia, and 8 (7.1%) as having confirmed sarcopenia.

The baseline demographic, clinical, nutritional, and disease-related characteristics of the study population are summarized in **Table 1**.

**Table 1:** Overall and sarcopenia-stratified characteristics of the study population according.

Variable	Overall (n=113)	Non sarcopenia (n=32)	Probable sarcopenia (n=73)	Confirmed sarcopenia (n=8)	p
Median age, y (IQR)	38.00 (26.00, 57.00)	51.00 [40.25, 61.25]	33.00 [25.00, 49.00]	24.00 [22.00, 33.50]	0.003
Male sex, n (%)	54 (47.8)	14 (43.8)	34 (46.6)	6 (75.0)	0.269
Surgery for IBD, n (%)	47 (41.6)	10 (31.2)	31 (42.5)	6 (75.0)	0.078
Crohn’s Disease, n (%)	65 (57.5)	15 (46.9)	44 (60.3)	6 (75)	0.258
Behavior, n (%)					0.670
B1	12 (18.5)	4 (26.7)	8 (18.2)	0 (0.0)	
B2	31 (47.7)	7 (46.7)	21 (47.7)	3 (50.0)	
B3	22 (33.8)	4 (26.7)	15 (34.1)	3 (50.0)	
Location, n (%)					0.085
L1	17 (26.2)	8 (53.3)	8 (18.2)	1 (16.7)	
L2	8 (12.3)	2 (13.3)	5 (11.4)	1 (16.7)	
L3	40 (61.5)	5 (33.3)	31 (70.5)	4 (66.7)	
Ulcerative colitis, n (%)	48 (42.5)	17 (53.1)	29 (39.7)	2 (25.0)	
Extension					0.527
E1	7 (14.6)	4 (23.5)	3 (10.3)	0 (0.0)	
E2	24 (50.0)	6 (35.3)	17 (58.6)	1 (50.0)	
E3	17 (35.4)	7 (41.2)	9 (31.0)	1 (50.0)	
Mean HBI score (SD)	5.95 (2.78)	4.73 (1.58)	6.09 (2.57)	8.00 (5.06)	0.041
Mean pMAYO score (SD)	4.69 (2.63)	4.29 (2.93)	4.83 (2.55)	6.00 (0.00)	0.629
Clinical activity, n (%)	85 (75.2)	23 (71.9)	56 (76.7)	6 (75.0)	0.834
Ultrasound activity, n (%)	55 (48.7)	12 (37.5)	39 (53.4)	4 (50.0)	0.322
Mean SES-CD (SD)	6.29 (5.62)	4.47 (2.53)	6.50 (5.83)	9.33 (8.52)	0.184
Mean MES (SD)	1.88 (0.87)	1.94 (0.83)	1.76 (0.87)	3.00 (0.00)	0.135
Endoscopic activity, n (%)	84 (74.3)	26 (81.2)	51 (69.9)	7 (87.5)	0.318
Median albumin, g/dL (IQR)	4.10 (3.80, 4.30)	4.10 [3.77, 4.22]	4.10 [3.80, 4.30]	3.95 [3.28, 4.40]	0.749
Median CRP, mg/L (IQR)	3.00 (2.00, 6.10)	2.44 [1.40, 4.03]	3.00 [2.00, 4.00]	9.50 [6.85, 19.35]	0.026
Median calprotectin, mcg/g (IQR)	89.00 (40.00, 372.00)	76.00 [30.00, 291.00]	99.00 [42.00, 371.00]	165.00 [92.50, 585.75]	0.594
Median adiponectin, μg/mL (IQR)	18.4 (14.821.9)	20.1 (16.65–23.10)	18.8 (16.70–21.30)	17.55 (10.63–22.55)	0.367
EIMs, n (%)	38 (33.6)	13 (40.6)	23 (31.5)	2 (25.0)	0.572
Type of EIMs, n (%)					0.566
Joint	31 (27.4)	12 (37.5)	18 (24.7)	1 (12.5)	
Skin	5 (4.4)	1 (3.1)	3 (4.1)	1 (12.5)	
Eye	2 (1.8)	0 (0.0)	2 (2.7)	0 (0.0)	
None	75 (66.4)	19 (59.4)	50 (68.5)	6 (75.0)	
Bio failure, n (%)	41 (36.3)	12 (37.5)	25 (34.2)	4 (50.0)	0.669
Advanced therapy, n (%)	63 (55.8)	20 (62.5)	39 (53.4)	4 (50.0)	0.651
Therapy type, n (%)					0.135
Anti-TNF-alpha	31 (27.4)	11 (34.4)	19 (26.0)	1 (12.5)	
JAKi	7 (6.2)	2 (6.2)	3 (4.1)	2 (25.0)	
UST	14 (12.4)	4 (12.5)	10 (13.7)	0 (0.0)	
VDZ	15 (13.3)	4 (12.5)	10 (13.7)	1 (12.5)	
Mean BMI, kg/m2 (SD)	24.54 (5.21)	29.21 (4.27)	23.01 (4.26)	19.80 (4.15)	<0.001
Median Handgrip, kg (IQR)	26.00 (19.00, 33.00)	26.00 (20.00, 36.00)	26.00 (18.00, 33.00)	22.50 (20.00, 25.0)]	0.272
Median FFMI, kg/m² (IQR)	18.16 (17.25, 19.90)	21.02 (19.47, 22.01)	17.81 (17.05, 18.75)	16.20 (15.43, 17.11)	<0.001
Mean IBD-DI (SD)	43.95 (12.09)	42.50 (11.16)	44.16 (11.69)	47.75 (18.86)	0.533
Mean IBD-Disk (SD)	40.35 (24.93)	35.69 (25.60)	42.19 (24.90)	42.25 (22.83)	0.461
Moderate-to-severe disability, n (%)	52 (46.0)	12 (37.5)	37 (50.7)	3 (37.5)	0.405
Mean SIBDQ (SD)	46.81 (12.25)	48.41 (11.96)	46.40 (12.41)	44.12 (12.82)	0.608
Malnutrition risk, n (%)	37 (32.7)	4 (12.5)	27 (37.0)	6 (75.0)	0.001
MEDI-lite adherence, N (%)	99 (87.6)	31 (96.9)	62 (84.9)	6 (75.0)	0.123

IQR, interquartile range; SD, standard deviation; IBD, inflammatory bowel disease; CD, Crohn’s disease; UC, ulcerative colitis; HBI, Harvey–Bradshaw Index; pMAYO, partial Mayo score; SES-CD, Simple Endoscopic Score for Crohn’s Disease; MES, Mayo Endoscopic Subscore; EIMs, extraintestinal manifestations; BMI, body mass index; IBD-DI, Inflammatory Bowel Disease Disability Index; IBD-Disk, Inflammatory Bowel Disease Disability Disk; SIBDQ, Inflammatory Bowel Disease Questionnaire; JAKi, Janus kinase inhibitors; UST, ustekinumab; VDZ, vedolizumab.

The median age was 38.0 years (IQR 26.0–57.0), and 59 patients (52.2%) were female. Age differed significantly across the three sarcopenia groups, with progressively lower median age from non-sarcopenic patients to those with probable and confirmed sarcopenia (51.0 [40.3–61.3], 33.0 [25.0–49.0], and 24.0 [22.0–33.5] years, respectively; p=0.003). Sex distribution, disease subtype, disease phenotype, and previous IBD-related surgery did not differ significantly across groups. No significant differences were observed in endoscopic or bowel ultrasound disease activity. Most clinical disease activity measures were also comparable among groups, although the HBI differed significantly across sarcopenia categories (p = 0.041).

In contrast, BMI differed significantly according to sarcopenia status. Patients with probable or confirmed sarcopenia had lower BMI than non-sarcopenic patients (29.21 ± 4.27 vs. 23.01 ± 4.26 vs. 19.80 ± 4.15 kg/m² for non-sarcopenic, probable, and confirmed sarcopenia, respectively; p < 0.001).

Among laboratory parameters, serum CRP differed significantly across groups. Median CRP concentrations were 2.44 mg/L (IQR 1.40–4.03) in non-sarcopenic patients, 3.00 mg/L (IQR 2.00–4.00) in patients with probable sarcopenia, and 9.50 mg/L (IQR 6.85–19.35) in those with confirmed sarcopenia (p = 0.026). After Holm correction, CRP was significantly higher in confirmed sarcopenia than in both non-sarcopenic (p = 0.020) and probable sarcopenia (p = 0.026), whereas no difference emerged between the latter groups (p = 0.528). Serum albumin concentrations were comparable across groups.

Although circulating adiponectin concentrations did not differ significantly (p = 0.367). Median adiponectin levels were 20.1 μg/mL (IQR 16.7–23.1) in non-sarcopenic patients, 18.8 μg/mL (IQR 16.7–21.3) in patients with probable sarcopenia, and 17.6 μg/mL (IQR 10.6–22.6) in those with confirmed sarcopenia. Nutritional risk was significantly associated with sarcopenia status. The proportion of patients at high risk of malnutrition increased from 12.5% in the non-sarcopenic group to 37.0% among patients with probable sarcopenia and 75.0% among those with confirmed sarcopenia (p = 0.001).

No statistically significant differences were observed among the three groups regarding disability scores, health-related quality of life, extraintestinal manifestations, biologic exposure, previous biologic failure, or current biologic therapy.

### Clinical outcomes

All patients completed the predefined 12-month follow-up. Overall, disease clinical worsening occurred in 55 patients (48.7%), IBD-related hospitalization in 15 (13.3%), IBD-related surgery in 9 (8.0%), infectious complications in 13 (11.5%), and treatment modification in 29 (25.7%).

Clinical worsening occurred in 46.9% of non-sarcopenic patients, 45.2% of patients with probable sarcopenia, and 87.5% of those with confirmed sarcopenia (p = 0.074).

IBD-related hospitalization was observed in 6.2%, 11.0%, and 62.5% of patients, respectively (p < 0.001).

The need for IBD-related surgery was observed in 3.1%, 6.8%, and 37.5% of patients across the non-sarcopenic, probable sarcopenia, and confirmed sarcopenia groups, respectively (p = 0.005). Likewise, infectious complications occurred in 6.2%, 11.0%, and 37.5% of patients, respectively (p = 0.045).

Following Holm correction for multiple pairwise comparisons, confirmed sarcopenia was associated with a significantly higher rate of IBD-related hospitalization compared with both non-sarcopenic patients (adjusted p = 0.0046) and patients with probable sarcopenia (adjusted p = 0.0046). No other pairwise comparison remained statistically significant after Holm correction (**Figure 2**).

**Figure 2:**
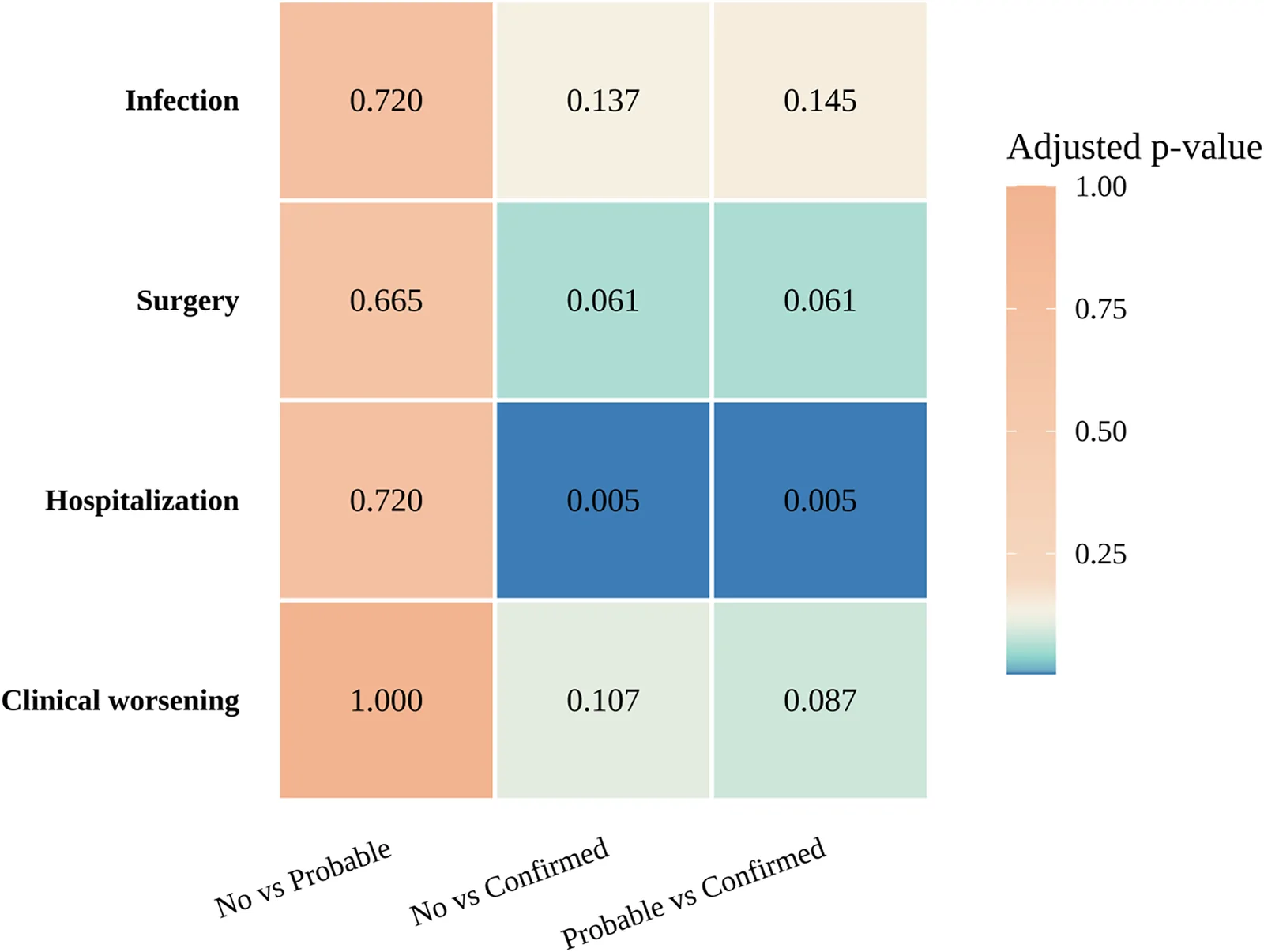
Holm-adjusted pairwise comparisons of adverse outcomes across sarcopenia groups.

Treatment modification occurred in 18.8% of non-sarcopenic patients, 23.3% of patients with probable sarcopenia, and 75.0% of those with confirmed sarcopenia (p = 0.004).

In a sensitivity analysis restricted to patients in clinical remission at baseline (n = 28), clinical worsening occurred in 55.6%, 17.6%, and 50.0% of patients without sarcopenia, with probable sarcopenia, and with confirmed sarcopenia, respectively, with no significant difference across groups (Fisher’s exact p = 0.082).

In a further sensitivity analysis restricted to patients in both clinical and endoscopic remission at baseline (n = 14), clinical worsening occurred in 80.0%, 25.0%, and 0% of patients without sarcopenia, with probable sarcopenia, and with confirmed sarcopenia, respectively, with no significant difference across groups (Fisher’s exact p = 0.091).

### Multivariable analysis

In multivariable analyses, neither probable nor confirmed sarcopenia was independently associated with clinical worsening after adjustment for age, baseline clinical activity, BMI, and CRP (probable sarcopenia: aOR 0.75, 95% CI 0.25–2.16; p=0.591; confirmed sarcopenia: aOR 3.82, 95% CI 0.45–52.04; p=0.227). Baseline clinical activity (aOR 3.14, 95% CI 1.21–9.02; p=0.017) and higher CRP levels (aOR 1.09 per mg/L, 95% CI 1.02–1.18; p=0.004) were independently associated with clinical worsening (**Figure 3A**). No relevant multicollinearity was detected in the main multivariable model (adjusted GVIF values, 1.03–1.26).

**Figure 3:**
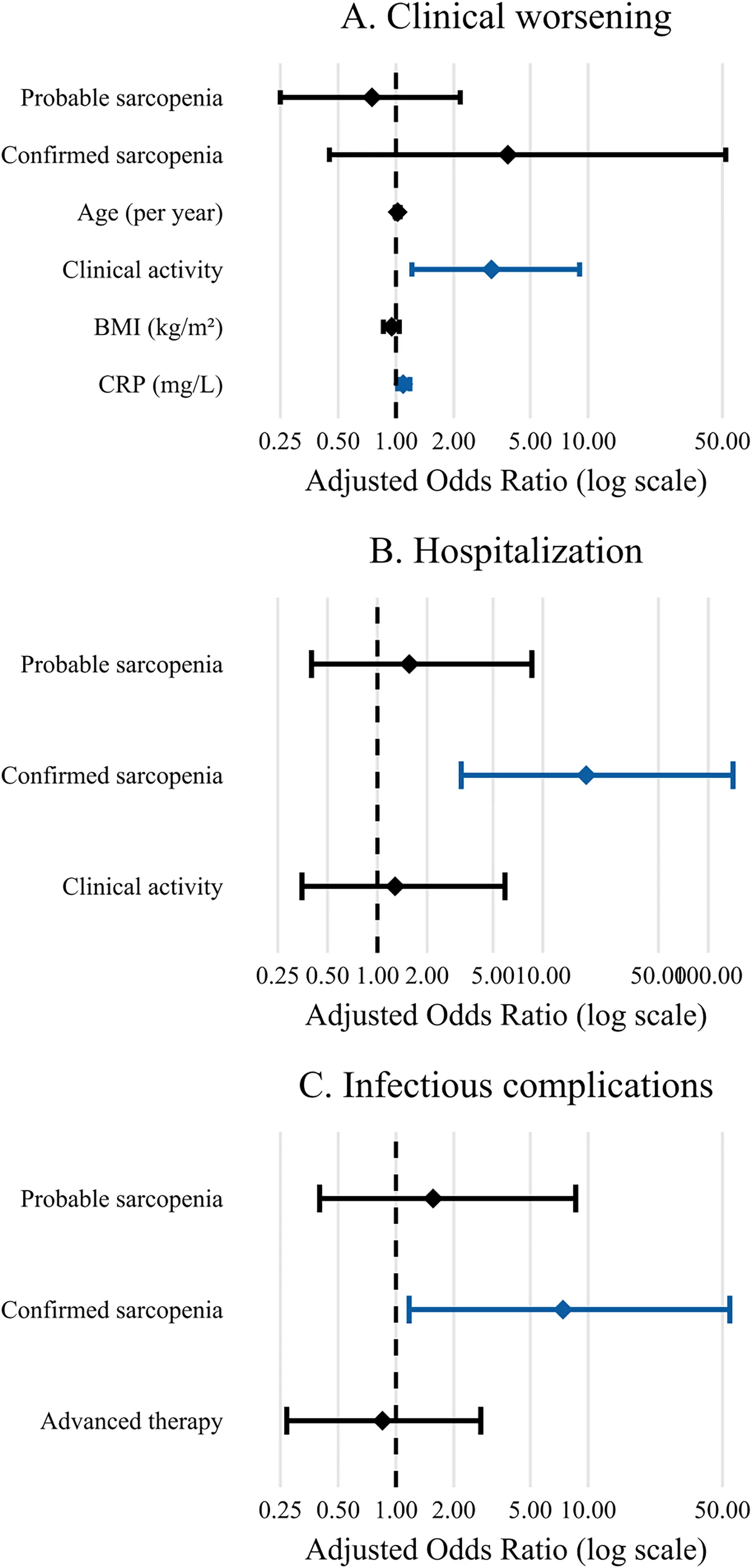
Multivariable logistic regression models for clinical worsening **(A)**, hospitalization **(B)**, and infections **(C)** according to sarcopenia status.

For IBD-related hospitalization, confirmed sarcopenia was independently associated with the outcome after adjustment for baseline clinical activity (aOR 18.30, 95% CI 3.21–141.14; p<0.001), whereas probable sarcopenia was not (aOR 1.56, 95% CI 0.40–8.58; p=0.544) (**Figure 3B**).

Similarly, confirmed sarcopenia was independently associated with infectious complications after adjustment for advanced therapy exposure (aOR 7.40, 95% CI 1.17–54.63; p=0.034), whereas probable sarcopenia was not (aOR 1.56, 95% CI 0.40–8.62; p=0.540). Advanced therapy exposure was not independently associated with infectious complications (aOR 0.85, 95% CI 0.27–2.76; p=0.780) (**Figure 3C**).

The apparent discriminative performance of the multivariable models was moderate, with an AUC of 0.722 (95% CI 0.628–0.816) for clinical worsening, 0.692 (95% CI 0.543–0.841) for hospitalization, and 0.636 (95% CI 0.484–0.789) for infectious complications.

Complete univariable and multivariable Firth penalized logistic regression analyses are reported in **Supplementary Table S1** and **S2**, respectively.

## Discussion

IBD is increasingly recognized as a systemic disorder extending beyond the gastrointestinal tract. Persistent inflammation, impaired nutrient intake and absorption, metabolic alterations, and reduced physical activity contribute to progressive changes in body composition, ultimately leading to skeletal muscle dysfunction (24–27).

In this prospective cohort study, we investigated the prevalence and prognostic relevance of sarcopenia in IBD patients, using a comprehensive assessment combining muscle strength and muscle mass according to the EWGSOP2 framework (4).

The prevalence of confirmed sarcopenia observed in our cohort was relatively low (7.1%), whereas probable sarcopenia was considerably more frequent (64.6%). The prevalence of diagnosis of sarcopenia in IBD varies widely across studies, largely because of differences in study populations, definitions, and methods used to quantify muscle mass and function. A systematic review by Fatani et al. estimated a prevalence of approximately 17% for sarcopenia, while highlighting substantial methodological heterogeneity across available studies. More recently, prospective cohorts applying both morphological and functional criteria have reported variable prevalence estimates, further emphasizing the importance of the diagnostic approach adopted. Importantly, much of the previous IBD literature has focused predominantly on radiological assessments of skeletal muscle mass, frequently using CT-derived skeletal muscle indices, rather than incorporating muscle function into the definition of sarcopenia (13, 14, 28–31).

The present study therefore adds to the available evidence by applying the EWGSOP2 conceptual framework, in which reduced muscle strength represents the initial hallmark of probable sarcopenia and the concomitant demonstration of reduced muscle mass establishes the diagnosis of confirmed sarcopenia (4).

A particularly noteworthy finding was that sarcopenia in our population cannot be interpreted merely as an age-related phenomenon. Indeed, patients with confirmed sarcopenia were remarkably young, with a median age of 24 years, compared with 51 years among patients without sarcopenia. Although this apparently paradoxical finding should be interpreted cautiously because only eight patients had confirmed sarcopenia, it reinforces the concept that muscle impairment in IBD may develop independently of physiological ageing (24, 27, 32).

Moreover, patients with confirmed sarcopenia had markedly lower BMI and higher CRP concentrations than patients without sarcopenia, while the prevalence of high nutritional risk progressively increased across muscle-status categories, reaching 75% among patients with confirmed sarcopenia. Interestingly, serum albumin concentrations did not differ across groups. These findings underline that conventional anthropometric or biochemical markers considered individually may not adequately characterize nutritional and metabolic vulnerability in IBD. BMI, in particular, provides limited information on body composition and cannot distinguish adipose tissue from metabolically and functionally relevant skeletal muscle. Muscle assessment may therefore offer complementary information beyond traditional nutritional screening (25, 26, 28, 33).

Chronic inflammation may represent a major mechanistic link between intestinal disease and skeletal muscle impairment. Persistent exposure to pro-inflammatory cytokines can promote proteolysis, impair muscle protein synthesis, and alter metabolic homeostasis, while nutritional deficiencies and reduced physical activity may further amplify muscle loss. This interaction is likely bidirectional and may contribute to the proposed gut–muscle axis, in which intestinal inflammation, alterations in nutrient absorption and metabolism, and changes in the gut microbiota collectively influence skeletal muscle homeostasis. In our cohort, CRP concentrations were substantially higher in patients with confirmed sarcopenia, supporting an association between systemic inflammatory burden and advanced muscle impairment. Nevertheless, the observational design of the study precludes establishing whether inflammation primarily drives sarcopenia, whether impaired muscle status contributes to systemic vulnerability, or whether both represent manifestations of a more severe underlying disease phenotype (24, 25, 27).

The most clinically relevant observation of the present study was the strong association between confirmed sarcopenia and IBD-related hospitalization. During follow-up, hospitalization occurred in 62.5% of patients with confirmed sarcopenia compared with 11.0% of those with probable sarcopenia and 6.2% of patients without sarcopenia. After adjustment for baseline clinical activity, confirmed sarcopenia remained independently associated with hospitalization, with an approximately 18-fold increase in the odds of this outcome. Although the magnitude of this estimate must be interpreted in light of the small number of patients with confirmed sarcopenia and the resulting wide confidence interval, the direction of the association is consistent with an increasing body of evidence linking impaired muscle status to adverse outcomes in IBD (29, 31, 34).

Previous studies examining the prognostic impact of sarcopenia have produced heterogeneous results, partly reflecting differences in definitions and outcome ascertainment. Systematic reviews have nevertheless suggested associations between reduced muscle mass or sarcopenia and treatment failure, hospitalization, surgery, and postoperative complications. More recent prospective data have also supported an association between sarcopenia and subsequent hospital admission. Our findings extend these observations by showing that an EWGSOP2-defined phenotype integrating both impaired muscle strength and reduced muscle mass may provide prognostic information beyond baseline clinical activity. This distinction may be clinically relevant, as muscle weakness alone was common in our cohort but was not associated with a comparable increase in adverse outcomes (13, 14, 29–31, 34).

Another relevant observation concerns infectious complications. Patients with confirmed sarcopenia experienced a substantially higher incidence of infections during follow-up, and confirmed sarcopenia remained independently associated with infectious complications after adjustment for exposure to advanced therapies. Of note, advanced therapy itself was not independently associated with infections in the corresponding model. Previous data from patients with active CD have similarly shown higher infection rates among sarcopenic individuals, and an association between sarcopenia and susceptibility to infection has also been described outside the IBD setting. Several mechanisms may contribute to this relationship. Skeletal muscle is increasingly recognized as an immunometabolically active organ, and severe muscle depletion may reflect impaired metabolic reserve, nutritional deficiencies, chronic inflammation, and altered immune competence. Therefore, sarcopenia may identify patients with reduced resilience to infectious stress independently of pharmacological immunosuppression. However, given the limited number of infectious events in our study, this finding should be considered hypothesis-generating and requires confirmation in larger prospective cohorts (31, 35).

In contrast to hospitalization and infections, sarcopenia was not independently associated with the broader endpoint of clinical worsening after adjustment for age, baseline clinical activity, BMI, and CRP. Baseline clinical activity and CRP were instead independently associated with this outcome. This finding should be considered informative rather than contradictory. Clinical worsening predominantly reflects intestinal inflammatory activity and may therefore be more directly captured by established markers of active disease. Sarcopenia, conversely, may represent a marker of global physiological vulnerability that becomes particularly relevant for harder clinical outcomes such as hospitalization or infection.

The higher crude rates of IBD-related surgery and treatment modification observed among patients with confirmed sarcopenia are also noteworthy. However, because of the small number of events and the limited size of the confirmed-sarcopenia group, robust multivariable modelling of these outcomes was not possible. These associations should consequently be interpreted as exploratory. Nevertheless, they are consistent with previous studies and meta-analyses suggesting that impaired skeletal muscle status may be associated with treatment failure and surgical outcomes in IBD and provide a rationale for further investigation in adequately powered prospective studies (15, 30, 31, 34, 36).

Our study has several strengths. Patients were consecutively enrolled and prospectively followed for 12 months, and adverse outcomes were prospectively recorded. Sarcopenia was assessed using both functional and quantitative measures according to the EWGSOP2 framework rather than relying exclusively on radiological estimates of muscle mass. Furthermore, the cohort was extensively characterized in terms of clinical and endoscopic activity, intestinal ultrasound, inflammatory biomarkers, nutritional risk, body composition, treatment exposure, disability, and quality of life. Finally, given the low number of events and the potential for sparse-data bias, Firth penalized logistic regression and parsimonious prespecified models were used to reduce small-sample bias and overfitting.

Several limitations should also be acknowledged. First, this was a single-center study with a relatively small sample size, and only eight patients fulfilled criteria for confirmed sarcopenia. Consequently, effect estimates for this group were characterized by wide confidence intervals and should not be interpreted as precise estimates of effect magnitude. Second, the limited number of hospitalization and infection events constrained the number of covariates that could be included in multivariable analyses, and residual confounding cannot be excluded. In particular, the associations observed cannot establish that sarcopenia itself causally increases the risk of adverse outcomes. Third, muscle mass was estimated using bioelectrical impedance analysis, which, although feasible and widely applicable in clinical practice, may be influenced by hydration status and is less precise than reference imaging-based techniques. Fourth, physical performance was not systematically assessed, precluding classification of severe sarcopenia according to EWGSOP2 criteria. Fifth, the high prevalence of active disease in the study population and the tertiary-care setting may limit generalizability to unselected community-based IBD populations or patients in sustained remission. Finally, sarcopenia was assessed at a single time point, preventing evaluation of longitudinal changes in muscle status and their relationship with disease control and subsequent outcomes (4, 23).

In conclusion, three main findings emerged from our analysis. First, impairment of muscle status was highly prevalent, with almost two-thirds of patients fulfilling criteria for probable sarcopenia, although confirmed sarcopenia was identified in a substantially smaller proportion of the cohort. Second, confirmed sarcopenia identified a subgroup of patients characterized by a distinct clinical and nutritional vulnerability, including lower BMI, higher systemic inflammatory burden, and a markedly greater prevalence of high nutritional risk. Third, and most importantly, confirmed sarcopenia was strongly associated with adverse outcomes during the subsequent 12 months, particularly IBD-related hospitalization and infectious complications, and these associations persisted after adjustment for selected clinically relevant confounders. Conversely, probable sarcopenia alone was not independently associated with these outcomes. Taken together, these findings suggest that the coexistence of reduced muscle strength and reduced muscle mass may identify a particularly vulnerable IBD phenotype that is not fully captured by conventional measures of disease activity or nutritional status.

## Conclusions

In conclusion, our prospective findings suggest that confirmed sarcopenia identifies a small but clinically vulnerable subgroup of IBD patients at substantially increased risk of hospitalization and infectious complications. Importantly, this association appears to extend beyond conventional measures of baseline disease activity and treatment exposure, whereas isolated impairment of muscle strength was not associated with the same adverse prognostic profile. These observations support the concept that skeletal muscle health may represent an additional dimension of risk stratification in IBD. Larger multicenter prospective studies are warranted to validate these findings, establish IBD-specific prognostic thresholds, determine the incremental predictive value of muscle assessment over established disease-activity markers, and ultimately investigate whether targeted interventions aimed at restoring muscle health can modify the clinical course of IBD.

## Data Availability

The raw data supporting the conclusions of this article will be made available by the authors, without undue reservation.

## Supplementary material

Supplementary Table 1 Univariable Firth penalized logistic regression analyses of factors associated with clinical outcomes.

Supplementary Table 2 Multivariable Firth penalized logistic regression analyses of factors associated with clinical deterioration, hospitalization, and infectious complications.

## References

[1] Kucharzik T Taylor S Allocca M Burisch J Ellul P Iacucci M . ECCO-ESGAR-ESP-IBUS guideline on diagnostics and monitoring of patients with inflammatory bowel disease: Part 1. J Crohns Colitis. (2025) 19:jjaf106. doi: 10.1093/ecco-jcc/jjaf106 40741688

[2] Kaplan GG . The global burden of IBD: from 2015 to 2025. Nat Rev Gastroenterol Hepatol. (2015) 12:720–7. doi: 10.1038/nrgastro.2015.150 26323879

[3] Pizzoferrato M de Sire R Ingravalle F Mentella MC Petito V Martone AM . Characterization of sarcopenia in an IBD population attending an Italian gastroenterology tertiary center. Nutrients. (2019) 11:2281. doi: 10.3390/nu11102281 31554166 PMC6835412

[4] Cruz-Jentoft AJ Bahat G Bauer J Boirie Y Bruyère O Cederholm T . Sarcopenia: revised European consensus on definition and diagnosis. Age Ageing. (2019) 48:16–31. doi: 10.1093/ageing/afy169 30312372 PMC6322506

[5] Forbes A Escher J Hébuterne X Kłęk S Krznaric Z Schneider S . ESPEN guideline: Clinical nutrition in inflammatory bowel disease. Clin Nutr. (2017) 36:321–47. doi: 10.1016/j.clnu.2016.12.027 28131521

[6] Nguyen GC Munsell M Harris ML . Nationwide prevalence and prognostic significance of clinically diagnosable protein-calorie malnutrition in hospitalized inflammatory bowel disease patients. Inflammation Bowel Dis. (2008) 14:1105–11. doi: 10.1002/ibd.20429 18302272

[7] Cioffi I Imperatore N Di Vincenzo O Pagano MC Santarpia L Pellegrini L . Evaluation of nutritional adequacy in adult patients with Crohn's disease: a cross-sectional study. Eur J Nutr. (2020) 59:3647–58. doi: 10.1007/s00394-020-02198-0 32072266 PMC7669764

[8] Cioffi I Marra M Imperatore N Pagano MC Santarpia L Alfonsi L . Assessment of bioelectrical phase angle as a predictor of nutritional status in patients with Crohn's disease: A cross sectional study. Clin Nutr. (2020) 39:1564–71. doi: 10.1016/j.clnu.2019.06.023 31303525

[9] Bahat G Tufan A Tufan F Kilic C Akpinar TS Kose M . Cut-off points to identify sarcopenia according to European Working Group on Sarcopenia in Older People (EWGSOP) definition. Clin Nutr. (2016) 35:1557–63. doi: 10.1016/j.clnu.2016.02.002 26922142

[10] Skrzypczak D Ratajczak AE Szymczak-Tomczak A Dobrowolska A Eder P Krela-Kaźmierczak I . A vicious cycle of osteosarcopenia in inflammatory bowel diseases-aetiology, clinical implications and therapeutic perspectives. Nutrients. (2021) 13:293. doi: 10.3390/nu13020293 33498571 PMC7909530

[11] Dhaliwal A Quinlan JI Overthrow K Greig C Lord JM Armstrong MJ . Sarcopenia in inflammatory bowel disease: A narrative overview. Nutrients. (2021) 13:656. doi: 10.3390/nu13020656 33671473 PMC7922969

[12] Ünal NG Oruç N Tomey O Ömer Özütemiz A . Malnutrition and sarcopenia are prevalent among inflammatory bowel disease patients with clinical remission. Eur J Gastroenterol Hepatol. (2021) 33:1367–75. doi: 10.1097/MEG.0000000000002044 33470696

[13] Fatani H Olaru A Stevenson R Alharazi W Jafer A Atherton P . Systematic review of sarcopenia in inflammatory bowel disease. Clin Nutr. (2023) 42:1276–91. doi: 10.1016/j.clnu.2023.05.002 37352818

[14] Ryan E McNicholas D Creavin B Kelly ME Walsh T Beddy D . Sarcopenia and inflammatory bowel disease: A systematic review. Inflammation Bowel Dis. (2019) 25:67–73. doi: 10.1093/ibd/izy212 29889230

[15] Grova M Crispino F Maida M Vitello A Renna S Casà A . Sarcopenia is a negative predictive factor for endoscopic remission in patients with Crohn's disease treated with biologics. Dig Liver Dis. (2023) 55:865–71. doi: 10.1016/j.dld.2023.02.017 36925319

[16] Nigro E Scudiero O Monaco ML Palmieri A Mazzarella G Costagliola C . New insight into adiponectin role in obesity and obesity-related diseases. BioMed Res Int. (2014) 2014:658913. doi: 10.1155/2014/658913 25110685 PMC4109424

[17] Nigro E Marra M De Santis F Sammarco R Leo C Nardone OM . Adiponectin and phase angle in the assessment of sarcopenia in Crohn's disease: beyond muscle mass. Front Nutr. (2026) 13:1753532. doi: 10.3389/fnut.2026.1753532 42111839 PMC13149123

[18] von Elm E Altman DG Egger M Pocock J Gøtzsche PC Vandenbroucke JP . Strengthening the reporting of observational studies in epidemiology (STROBE) statement: guidelines for reporting observational studies. BMJ. (2007) 335:806–8. doi: 10.1136/bmj.39335.541782.AD 17947786 PMC2034723

[19] Shafer LA Shaffer S Witt J Nugent Z Bernstein CN . IBD Disability Index is associated with both direct and indirect costs of inflammatory bowel disease. Inflammation Bowel Dis. (2022) 28:1189–97. doi: 10.1093/ibd/izab248 34636400

[20] Chao PC Chuang HJ Tsao LY Chen PY Hsu CF Lin HC . The Malnutrition Universal Screening Tool (MUST) and a nutrition education program for high risk cancer patients: strategies to improve dietary intake in cancer patients. Biomedicine (Taipei). (2015) 5:17. doi: 10.7603/s40681-015-0017-6 26264480 PMC4532677

[21] Sandhu A Mosli M Yan B Wu T Gregor J Chande N . Self-screening for malnutrition risk in outpatient inflammatory bowel disease patients using the Malnutrition Universal Screening Tool (MUST). JPEN J Parenter Enteral Nutr. (2016) 40:507–10. doi: 10.1177/0148607114566656 25632031

[22] Dinu M Pagliai G Lotti S Giangrandi I Colombini B Sofi F . Adherence to Mediterranean diet measured through Medi-Lite score and obesity: A retrospective study. Nutrients. (2021) 13:2007. doi: 10.3390/nu13062007 34200879 PMC8230530

[23] Sergi G De Rui M Veronese N Bolzetta F Berton L Carraro S . Assessing appendicular skeletal muscle mass with bioelectrical impedance analysis in free-living Caucasian older adults. Clin Nutr. (2015) 34:667–73. doi: 10.1016/j.clnu.2014.07.010 25103151

[24] Nardone OM de Sire R Petito V Testa A Villani G Scaldaferri F . Inflammatory bowel diseases and sarcopenia: The role of inflammation and gut microbiota in the development of muscle failure. Front Immunol. (2021) 12:694217. doi: 10.3389/fimmu.2021.694217 34326845 PMC8313891

[25] Massironi S Viganò C Palermo A Pirola L Mulinacci G Allocca M . Inflammation and malnutrition in inflammatory bowel disease. Lancet Gastroenterol Hepatol. (2023) 8:579–90. doi: 10.1016/S2468-1253(23)00011-0 36933563

[26] Balestrieri P Ribolsi M Guarino MPL Emerenziani S Altomare A Cicala M . Nutritional aspects in inflammatory bowel diseases. Nutrients. (2020) 12:372. doi: 10.3390/nu12020372 32023881 PMC7071234

[27] Nishikawa H Nakamura S Miyazaki T Kakimoto K Fukunishi S Asai A . Inflammatory bowel disease and sarcopenia: Its mechanism and clinical importance. J Clin Med. (2021) 10:4214. doi: 10.3390/jcm10184214 34575326 PMC8470813

[28] Bezzio C Brinch D Ribaldone DG Cappello M Ruzzon N Vernero M . Prevalence, risk factors and association with clinical outcomes of malnutrition and sarcopenia in inflammatory bowel disease: A prospective study. Nutrients. (2024) 16:3983. doi: 10.3390/nu16233983 39683376 PMC11643262

[29] Dermine S Bazin T Hassan FA Bettolo J Billiauws L Bourdillel J . Prevalence and impact of sarcopenia in patients with inflammatory bowel diseases: A prospective cohort study. Clin Res Hepatol Gastroenterol. (2025) 49:102555. doi: 10.1016/j.clinre.2025.102555 40021084

[30] Potcovaru CG Filip PV Neagu OM Diaconu LS Salmen T Cinteză D . Diagnostic criteria and prognostic relevance of sarcopenia in patients with inflammatory bowel disease-a systematic review. J Clin Med. (2023) 12:4713. doi: 10.3390/jcm12144713 37510827 PMC10381373

[31] Nardone OM Ponsiglione A de Sire R Calabrese G Liuzzi R Testa A . Impact of sarcopenia on clinical outcomes in a cohort of Caucasian active Crohn's disease patients undergoing multidetector CT-enterography. Nutrients. (2022) 14:3460. doi: 10.3390/nu14173460 36079718 PMC9458031

[32] Neelam PB Sharma A Sharma V . Sarcopenia and frailty in inflammatory bowel disease: Emerging concepts and evidence. JGH Open. (2024) 8:e13033. doi: 10.1002/jgh3.13033 38283070 PMC10821747

[33] Zhang Y Zhang L Gao X Dai C Huang Y Wu Y . Impact of malnutrition and sarcopenia on quality of life in patients with inflammatory bowel disease: A multicentre study. J Cachexia Sarcopenia Muscle. (2023) 14:2663–75. doi: 10.1002/jcsm.13341 37779327 PMC10751433

[34] Bedard K Rajabali N Tandon P Abraldes JG Peerani F . Association between frailty or sarcopenia and adverse outcomes in inflammatory bowel disease: A systematic review. Gastro Hep Adv. (2022) 1:241–50. doi: 10.1016/j.gastha.2021.11.009 39131118 PMC11307438

[35] Nelke C Dziewas R Minnerup J Meuth SG Ruck T . Skeletal muscle as potential central link between sarcopenia and immune senescence. EBioMedicine. (2019) 49:381–8. doi: 10.1016/j.ebiom.2019.10.034 31662290 PMC6945275

[36] Karsegard VL Ferlay O Maisonneuve N Kyle UG Dupertuis YM Genton L . Outil de dépistage simplifié de la dénutrition: Malnutrition Universal Screening Tool (MUST) [Simplified malnutrition screening tool: Malnutrition Universal Screening Tool (MUST). Rev Med Suisse Romande. (2004) 124:601–5 15573502

