# Supplementary material for: The hidden burden of sarcopenia in inflammatory bowel disease: a strong predictor of complications
Source: Front Gastroenterol (Lausanne). 2026 Oct 28;5:1960689. doi: 10.3389/fgstr.2026.1960689 (PMC13751511; doi:10.3389/fgstr.2026.1960689)
Supplement: Supplementary file 1 [file Table1.docx]

**Supplementary Table S1.** Univariable Firth penalized logistic regression analyses of factors associated with clinical outcomes.

| **Outcome** | **Variable** | **OR (95% CI)** | **p-value** |
| --- | --- | --- | --- |
| Clinical worsening | Probable sarcopenia | 0.93 (0.41–2.14) | 0.871 |
|  | Confirmed sarcopenia | 5.65 (1.05–57.96) | 0.043 |
|  | Age, per year | 1.01 (0.99–1.03) | 0.391 |
|  | Male sex | 0.35 (0.16–0.75) | 0.006 |
|  | Clinical activity | 2.42 (1.02–6.06) | 0.045 |
|  | BMI, kg/m² | 0.96 (0.89–1.03) | 0.221 |
|  | CRP, mg/L | 1.09 (1.03–1.17) | 0.001 |
|  | High risk of malnutrition | 1.88 (0.86–4.20) | 0.112 |
|  | Advanced therapy | 0.51 (0.24–1.08) | 0.079 |
|  | SIBDQ score | 0.95 (0.92–0.98) | 0.002 |
| Hospitalization | Probable sarcopenia | 1.58 (0.41–8.75) | 0.528 |
|  | Confirmed sarcopenia | 19.17 (3.31–149.95) | <0.001 |
|  | Age, per year | 0.96 (0.92–1.00) | 0.031 |
|  | Male sex | 2.34 (0.80–7.58) | 0.123 |
|  | Clinical activity | 1.24 (0.38–5.14) | 0.737 |
|  | BMI, kg/m² | 0.77 (0.64–0.89) | <0.001 |
|  | CRP, mg/L | 1.05 (1.01–1.09) | 0.012 |
|  | High risk of malnutrition | 2.67 (0.91–8.01) | 0.073 |
|  | Advanced therapy | 1.38 (0.47–4.47) | 0.566 |
|  | SIBDQ score | 0.99 (0.95–1.04) | 0.742 |
| Infections | Probable sarcopenia | 1.58 (0.41–8.75) | 0.528 |
|  | Confirmed sarcopenia | 7.76 (1.23–57.66) | 0.030 |
|  | Age, per year | 1.00 (0.96–1.03) | 0.959 |
|  | Male sex | 0.20 (0.04–0.73) | 0.013 |
|  | Clinical activity | 3.12 (0.70–29.57) | 0.150 |
|  | BMI, kg/m² | 0.90 (0.78–1.02) | 0.121 |
|  | CRP, mg/L | 1.04 (1.00–1.08) | 0.030 |
|  | High risk of malnutrition | 8.02 (2.40–33.74) | <0.001 |
|  | Advanced therapy | 0.77 (0.25–2.45) | 0.653 |
|  | SIBDQ score | 0.95 (0.91–1.00) | 0.050 |
| Surgery | Probable sarcopenia | 1.69 (0.32–16.94) | 0.565 |
|  | Confirmed sarcopenia | 13.36 (1.80–159.74) | 0.011 |
|  | Age, per year | 0.85 (0.71–0.94) | <0.001 |
|  | Male sex | 2.16 (0.58–9.50) | 0.253 |
|  | Clinical activity | 1.01 (0.25–5.68) | 0.987 |
|  | BMI, kg/m² | 0.75 (0.59–0.91) | 0.002 |
|  | CRP, mg/L | 1.04 (1.00–1.08) | 0.052 |
|  | High risk of malnutrition | 1.75 (0.44–6.56) | 0.410 |
|  | Advanced therapy | 1.31 (0.35–5.77) | 0.690 |
|  | SIBDQ score | 1.01 (0.96–1.07) | 0.701 |

**Supplementary Table S2.** Multivariable Firth penalized logistic regression analyses of factors associated with clinical deterioration, hospitalization, and infectious complications.

| **Outcome** | **N** | **Events** | **Variable** | **Adjusted OR (95% CI)** | **p-value** |
| --- | --- | --- | --- | --- | --- |
| Clinical worsening | 113 | 55 | Probable sarcopenia | 0.75 (0.25–2.16) | 0.591 |
|  | 113 | 55 | Confirmed sarcopenia | 3.82 (0.45–52.04) | 0.227 |
|  | 113 | 55 | Age, per year | 1.02 (0.99–1.05) | 0.188 |
|  | 113 | 55 | Clinical activity | 3.14 (1.21–9.02) | 0.017 |
|  | 113 | 55 | BMI, kg/m² | 0.95 (0.86–1.04) | 0.250 |
|  | 113 | 55 | CRP, mg/L | 1.09 (1.02–1.18) | 0.004 |
| Hospitalization | 113 | 15 | Probable sarcopenia | 1.56 (0.40–8.58) | 0.544 |
|  | 113 | 15 | Confirmed sarcopenia | 18.30 (3.21–141.14) | <0.001 |
|  | 113 | 15 | Clinical activity | 1.28 (0.35–5.91) | 0.724 |
| Infections | 113 | 13 | Probable sarcopenia | 1.56 (0.40–8.62) | 0.540 |
|  | 113 | 13 | Confirmed sarcopenia | 7.40 (1.17–54.63) | 0.034 |
|  | 113 | 13 | Advanced therapy | 0.85 (0.27–2.76) | 0.780 |
